# Fault Diagnosis for Analog Circuits by Using EEMD, Relative Entropy, and ELM

**DOI:** 10.1155/2016/7657054

**Published:** 2016-09-08

**Authors:** Jian Xiong, Shulin Tian, Chenglin Yang

**Affiliations:** ^1^School of Automation Engineering, University of Electronic Science and Technology of China, Chengdu 611731, China; ^2^Department of Communication Engineering, Chengdu Technological University, Chengdu 611731, China

## Abstract

This paper presents a novel fault diagnosis method for analog circuits using ensemble empirical mode decomposition (EEMD), relative entropy, and extreme learning machine (ELM). First, nominal and faulty response waveforms of a circuit are measured, respectively, and then are decomposed into intrinsic mode functions (IMFs) with the EEMD method. Second, through comparing the nominal IMFs with the faulty IMFs, kurtosis and relative entropy are calculated for each IMF. Next, a feature vector is obtained for each faulty circuit. Finally, an ELM classifier is trained with these feature vectors for fault diagnosis. Via validating with two benchmark circuits, results show that the proposed method is applicable for analog fault diagnosis with acceptable levels of accuracy and time cost.

## 1. Introduction

Numerous researches have indicated that analog circuit fault diagnosis is a significant fundamental for design validation and performance evaluation in the integrated circuit manufacturing fields [1–3]. In contrast to the well-developed diagnostic methods for digital circuits, diagnosis for analog circuits is an extremely difficult problem and an active research due to the following reasons: (1) there is lack of a reliable and practical fault modeling method for analog circuits because of the complexity and variability of analog circuit structures; (2) the parameter values of analog components are continuous; (3) the impact of tolerance and nonlinear nature issues cannot be ignored; (4) for actual analog circuits, test points are limitations.

The procedure of fault diagnosis for analog circuits can be generally classified into four stages: data acquisition, feature extraction, fault detection, and fault identification and isolation. As one of the foremost stages in fault diagnosis, feature extraction methods are closely related to the efficiency of fault diagnosis. Many feature extraction methods have been proposed such as correlation function technique [4], information entropy approach [5], the fast Fourier transform technique [6], and the wavelet transform technique [7]. Zhang et al. [8] directly used the output voltage as features for fault diagnosis of analog circuits without preprocessing methods, and the results of fault diagnosis are not very good. M. Aminian and F. Aminian proposed a diagnostic method of analog circuits using wavelet decomposition coefficients, principal component analysis (PCA), and data normalization to construct fault feature vectors and then trained and tested neural network classifiers [3]. The method can obtain higher accuracy of diagnosis. In [9], Long et al. adopted conventional time-domain feature vectors to train and test least squares support vector machines (LS-SVM) for fault diagnosis of analog circuits which has better accuracy than that with traditional wavelet feature vectors. For information entropy techniques, it is more sensitive to parameter variations of components in CUTs. Therefore, information entropy is widely used with other techniques for fault diagnosis [5, 10–12]. Xie et al. diagnosed soft faults of analog circuits using Rényi's entropy and the result is effective [5]. In [11], authors have developed a new fault diagnosis approach by using kurtosis and entropy of sampled signals as feature vectors to train a neural network classifier.

However, there are some problems which should be considered and solved in feature extraction. Firstly, how to select features to train classifiers should be considered because different features with different classifiers for analog fault diagnosis have different results. Secondly, we find that most of the aforementioned methods were validated with some discrete simulations data. That is, they only considered a CUT to be faulty when a component value is higher or lower than its nominal value by 50%. It means this method has low fault coverage. Thirdly, some methods should take the influence of tolerance and the continuity of faulty parameters into account.

In our work, therefore, we use the techniques of EEMD, kurtosis, and relative entropy to construct new feature vectors to train an ELM classifier to improve the diagnosability and reduce time cost. As an adaptive time frequency data analysis method ensemble empirical mode decomposition (EEMD) is suitable for linear, nonlinear, and no-stationary signals [13]. Recently, it has been successfully applied to extract significant fault features in many fields such as rotating machinery and locomotive roller bearings fault diagnosis [13–15]. Relative entropy method is rarely used in the analogy circuit fault diagnosis field. The difference between the probability distributions of faulty and fault-free circuits can be distinguished clearly by adopting relative entropy, because when a component is varied, the energy distribution is also changed which leads to change in relative entropy. Kurtosis is a measure of heavy tailed distribution of a real valued random variable. It can clearly describe the difference from waveforms. As a result, the combinational methods of kurtosis and relative entropy are suitable as fault features for analog fault diagnosis.

As a consequence, in this paper, we decomposed impulse responses of a CUT into IMFs using EEMD method and then adopting kurtosis and relative entropy techniques to obtain feature vectors. These features vectors can be used for diagnosis of faulty components among various variation possibilities. For this purpose, a classifier is needed. We selected extreme learning machine (ELM) classifier because it is proven to have excellent generalization performance and low computational cost [16, 17] when it is fed to train and test with fault features. Utilizing the combination of EEMD, relative entropy, and ELM algorithms for feature extraction and classification we can complete analog circuit fault diagnosis. It demonstrates reliable and accurate fault diagnosis with reduced test time.

This paper is organized as follows: Section 2 briefly presents the principle of EEMD, relative entropy, and ELM algorithms. In Section 3, the diagnostic procedure of the proposed method is introduced.  Section 4 shows the simulation experiment details and results for two benchmark analog circuits. And then the performance of the proposed method is also discussed in the Section. Finally the conclusions are drawn in Section 5.

## 2. A Review of Fundamental Theory

In the work, we combined EEMD, relative entropy, and ELM to perform fault diagnosis of analog circuits. Fundamentals of EEMD, relative entropy, and ELM are introduced firstly as follows.

### 2.1. Ensemble Empirical Mode Decomposition (EEMD)

Ensemble empirical mode decomposition, based on empirical mode decomposition (EMD), is to solve the aliasing in time frequency distribution with Gaussian white noise [13]. Based on simple assumption any signal consists of different simple intrinsic modes of oscillations from low to high frequency [13, 19]. Thus, original signal is defined as

(1) $$\begin{array}{c} x\left(\;t\;\right)=\overset{n}{\underset{i=1}{\sum }}c_{i}\left(\;t\;\right)+r_{n}\left(\;t\;\right), \end{array}$$

where *c*_*i*_(*t*) is the intrinsic mode functions (IMF). An IMF is defined as a simple oscillatory function that satisfies two conditions [18]:

1. It has the same number of extrema and zero crossing or has the difference no more than one between them.
2. The mean value of the envelopes defined by the local maxima and minima is zero.

From (1), we can see that the original signal is decomposed into *n* IMFs and one residue *r*_*n*_(*t*). The procedure of decomposition with shifting method is described as follows.

**Step 1 .**

Given a signal *x*(*t*), all local maxima and minima of it are gained firstly. Then upper and lower envelopes of the given signal are determined from a cubic spline interpolation of the local maxima and minima. Let *m*_1_ be the mean of the two envelopes and the first component *h*_1_(*t*) is obtained as

(2) $$\begin{array}{c} h_{1}\left(\;t\;\right)=x\left(\;t\;\right)-m_{1}. \end{array}$$

**Step 2 .**

Let *m*_11_ be the mean of *h*_1_(*t*)'s upper and lower envelopes and *h*_11_(*t*) is calculated as follows:

(3) $$\begin{array}{c} h_{11}\left(\;t\;\right)=h_{1}\left(\;t\;\right)-m_{11}. \end{array}$$

**Step 3 .**

Repeat the above procedure *n* times until *h*_1*n*_(*t*) satisfies IMF conditions. The first IMF *c*_1_(*t*) is obtained by *c*_1_(*t*) = *h*_1*n*_(*t*).

**Step 4 .**

Subtract *c*_1_(*t*) from *x*(*t*), and a residue is obtained as

(4) $$\begin{array}{c} r_{1}\left(\;t\;\right)=x\left(\;t\;\right)-c_{1}\left(\;t\;\right). \end{array}$$

**Step 5 .**

The residue, which contains useful information, is considered as main signal and Steps 1–4 are repeated to gain other IMFs. Formula (4) is rewritten as

(5) $$\begin{array}{c} r_{i}\left(\;t\;\right)=r_{i-1}\left(\;t\;\right)-c_{i}\left(\;t\;\right)\;i=1,2,\ldots ,n. \end{array}$$

**Step 6 .**

When the residue *r*_*n*_(*t*) becomes monotonic slope or has only one extreme, the whole procedure is stopped.

From the procedure, we can see that IMFs represent the degree of oscillation of signal in amplitude and frequency. It means that these IMFs contain much time frequency information of the signal. Thus, the authors in [13] indicated that the algorithm is a new high-performance signal processing approach which can deal with linear, nonlinear, and no-stationary signals. More details about this technique can be found in [13, 19].

### 2.2. Relative Entropy

Let *X* be a continuous random variable. *p*(*x*) and *q*(*x*) are the probability distributions of *X*. Relative entropy describes the distance between two probability distributions of *X*. The relative entropy is calculated as

(6) $$\begin{array}{c} D\left(\;p||q\;\right)=\overset{}{\underset{x\in X}{\int }}p\left(\;x\;\right)\log\frac{p\left(x\right)}{q\left(x\right)}, \end{array}$$

where *p*(*x*) denotes energy probability distribution function (PDF) of response voltages for faulty CUT and *q*(*x*) indicates normal response voltage PDF of fault-free CUT. When parameters of one or more components of CUT are changed, the PDF of corresponding output voltage will also vary. This means that it is more sensitive to parameter variations of components in CUT. By calculating the relative entropy between faulty and fault-free circuit, faults can be detected. Consequently, for fault diagnosis, relative entropy is suitable as fault feature.

### 2.3. Extreme Learning Machine

**Figure 1:**
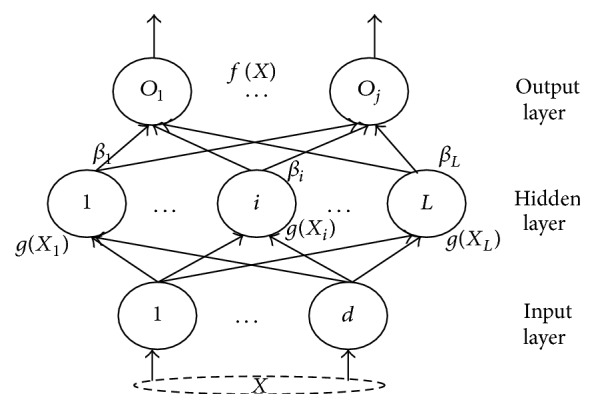
SLFN.

In order to accurately and quickly diagnose faults, in our work, extreme learning machine (ELM) is adopted. ELM is one kind of fast algorithm of single hidden-layer feedforward networks (SLFN) as shown in Figure 1. The hidden layer of SLFN need not be tuned. It is proven that it has excellent generalization performance and low computational cost in many applications [16, 17]. In the paper we utilize it to do fault diagnosis as a classifier. A brief of review of ELM is described as follows [16].

Suppose (*X*_*i*_, *t*_*i*_) ∈ *R* ^*n*^ × *R* ^*m*^ are *N* arbitrary distinct samples, *X*_*i*_ = [*x*_*i*1_, *x*_*i*__2_,…, *x*_*in*_]^*T*^. For a SLFN with *L* hidden nodes, taking one output node as example, the output function is defined as

(7) fLX=Oj=∑i=1LβigWi·Xj+bi=Hβ.j=1,2,…,N,

where *β* = [*β*_1_, *β*_2_,…, *β*_*L*_]^*T*^ is the output weight between hidden layer and output layer. *g*(*X*) is the activation function which demonstrates the output vector of the hidden layer with respect to the input *X*. *W*_*i*_ = [*w*_*i*1_, *w*_*i*2_,…, *w*_*in*_]^*T*^ is the input weight of the *i*th hidden node and *b*_*i*_ denotes the bias of hidden node *i*. And *W*_*i*_ · *X*_*j*_ represents the dot-product between *W*_*i*_ and *X*_*j*_. *H* is the hidden-layer output matrix. It is

(8) $$\begin{array}{c} H=\left[\begin{array}{ccc} g\left(W_{1}\cdot X_{1}+b_{1}\right) & \cdots & g\left(W_{L}\cdot X_{1}+b_{L}\right)\\ g\left(W_{1}\cdot X_{2}+b_{1}\right) & \cdots & g\left(W_{L}\cdot X_{2}+b_{L}\right)\\ \vdots & \cdots & \vdots \\ g\left(W_{1}\cdot X_{N}+b_{1}\right) & \cdots & g\left(W_{L}\cdot X_{N}+b_{L}\right) \end{array}\right]. \end{array}$$

The target of ELM is to minimize the output error; hence the minimal norm least square method is adopted.

(9) Minimize: Hβ−T2,

where *T* = [*t*_1_, *t*_2_ …, *t*_*N*_]^*T*^ indicates the expected value of output. Once *W*_*i*_ and *b*_*i*_ are determined, *H* is also uniquely confirmed. According to formula (7), the output weight can be calculated by

(10) $$\begin{array}{c} \beta =H^{+}T, \end{array}$$

where *H* ^+^ is the Moore–Penrose generalized inverse of matrix *H*.

## 3. Diagnostic Procedure

### 3.1. Diagnostic Procedure

**Figure 2:**
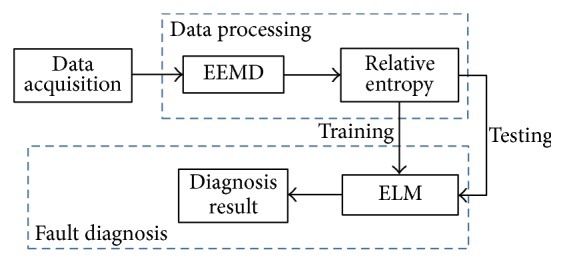
Block diagram of fault diagnosis.

The diagnostic procedure based on EEMD, relative entropy, and ELM is shown in Figure 2. The procedure of the proposed method involves four major stages: data acquisition, data processing, training, and fault diagnosis. Once the response voltage waveforms of fault-free circuit and fault circuits are recorded, respectively, they will be decomposed into IMF components by using EEMD. Through utilizing the energy of each IMF, then, kurtosis and relative entropy can be obtained between faulty IMFs and fault-free IMFs. Kurtosis and relative entropy of some IMFs of each fault are selected to compose a fault feature vector. The unique feature vector is extracted for each fault which is used for training and testing ELM classifier to complete fault diagnosis.

### 3.2. The Procedure of Feature Extraction

The procedure of feature extraction of the proposed method is described as follows.

**Step 1 .**

Every fault (including fault-free status) of CUT is simulated in PSPICE. And the relevant output waveforms are obtained.

**Step 2 .**

Decompose each waveform with EEMD into *n* IMFs according to the method in Section 2.1.

**Step 3 .**

Calculate kurtosis and relative entropy of each IMF.

*Step 3.1.* Obtain kurtosis from each IMF. According to [11], kurtosis is a measure of the heaviness of the tails in a distribution of the signal *x* [20]; hence, kurtosis could react to the change of signals and be used as feature of signals. Kurtosis is defined in the zero-mean case as follows [11]:

(11) $$\begin{array}{c} {kurt}_{i}\left(\;x\;\right)=E\left\{\;x^{4}\;\right\}-3{\left[\;E\left\{\;x^{2}\;\right\}\;\right]}^{2}\;i=1,2,\ldots ,n, \end{array}$$

where *E*() is the expectation operator; kurt_*i*_() is the kurtosis of IMF *i* for a fault. 

*Step 3.2.* Calculate relative entropy of each IMF.

**Figure 3:**
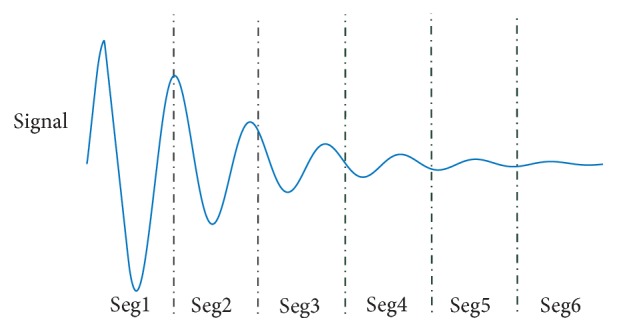
Segments of IMF.

(1) Calculate total energy of each IMF by
  (12) $$\begin{array}{c} E_{n}=\overset{K}{\underset{i=1}{\sum }}{\left(\;C_{n}\left(\;i\;\right)\;\right)}^{2}\;i=1,2,\ldots ,K, \end{array}$$
 where *C*_*n*_(*i*) is the *n*th IMF and the length of *C*_*n*_(*i*) is equal to *K*.
(2) Calculate probability distribution. According to [21], the nonnegative energy distribution can be visualized as probability distribution of signal. Hence, the process of calculating energy distribution is as follows: each IMF is averagely divided into *m* segments as shown in Figure 3 where *m* is 6. The energy of each segment is equal to
  (13) $$\begin{array}{c} E_{nm}=\overset{n_{2}}{\underset{i=n_{1}}{\sum }}{\left(\;C_{n}\left(\;i\;\right)\;\right)}^{2}, \end{array}$$
 where *m* = 1,2, 3,…, 6 is the number of segments; *n*_1_ and *n*_2_ are starting and stopping time points of the segment. The energy distribution of each segment in the whole IMF can be expressed as
  (14) $$\begin{array}{c} p_{nm}=\frac{E_{nm}}{E_{n}}. \end{array}$$
(3) According to the relative entropy theory, the definition of relative entropy of each IMFs is
  (15) $$\begin{array}{c} D_{n}=\overset{6}{\underset{m=1}{\sum }}p_{nm}\log\frac{p_{nm}}{p_{om}}, \end{array}$$
 where *p*_*om*_ is the energy distribution of segment of nominal IMF of fault-free circuit.

**Step 4 .**

A feature vector for each fault can be given as

(16) Tk=kurt1,kurt2,…,kurti,D1,D2,…,Dii=1,2,…,n,

where *k* denotes the number of fault samples in a circuit and *n* is the number of IMFs of one fault. Normalizing the feature vectors in formula (16) is reasonable to do. Here, we use partly normalized method to normalize some features in the feature vector which is defined as follows:

(17) Tnorm k=kurt1maxk⁡kurt1,kurt2maxk⁡kurt2,…,kurtimaxk⁡kurti,D1,D2,…,Dii=1,2,…,n.

Finally, we could use the feature vectors to train and test an ELM classifier for fault diagnosis.

## 4. Experiment and Performance Results

### 4.1. A Sallen-Key Bandpass Filter

**Figure 4:**
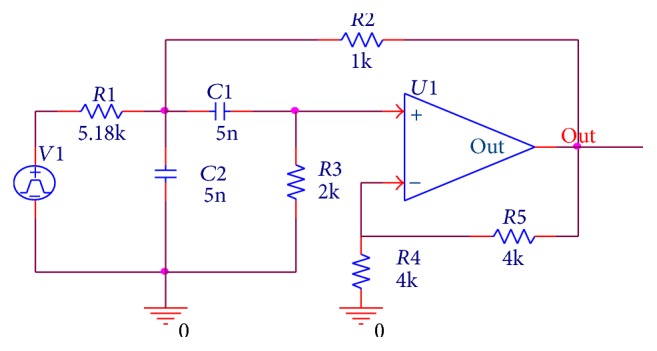
Second-order Sallen-Key bandpass filter circuit.

To verify the capacity of fault diagnosis with the proposed method, the first example circuit is a second-order Sallen-Key bandpass filter circuit, which is a benchmark circuit and is used as a CUT in [3, 9, 18].  Figure 4 shows the schematic of the circuit with nominal parameter values. From the figure, we can see that the filter circuit consists of 5 resistors, 2 capacitors, and 1 operational amplifier. First, the operational amplifiers in the circuit are assumed to be fault-free. Second we suppose each potential faulty component's nominal value is k and its faulty parameter range is [10^−4^ *∗*k, 95%*∗*k] and [105%*∗*k, 10^4^ *∗*k]. The nominal and faulty parameter ranges of the filter's components are shown in Table 1. In the table, there are total 15 faults including fault-free status where ⇑ and ⇓ stand for being higher and lower than nominal values, respectively.

According to the fault classes in Table 1, we use OrCAD/PSpice to simulate the circuit with time-domain transient analysis and Monte Carlo analysis methods to obtain the simulation fault data. First, the Sallen-Key bandpass filter is stimulated by a excitation signal* V1* which is a single pulse of 10 V with 10 *μ*s duration. The run to time and max step size are set as 300 *μ*s and 0.1 *μ*s, respectively. The output voltage values are gained at the point “out.” And, to consider the effects of the component tolerances, the resistors and capacitors are assumed to have tolerance limits of ±5%. When all the components are varying within their tolerances the circuit is considered no-fault. Otherwise, the parameter value of any component is out of scope of its tolerance limit with the other components varying within their tolerances which is regarded as a fault.

**Figure 5:**
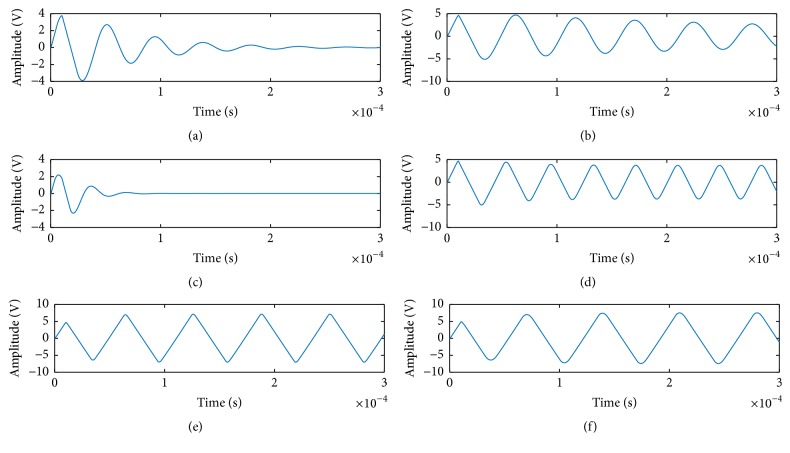
Examples of waveforms of fault-free status and fault status for the Sallen-Key bandpass filter circuit. (a) It is fault-free waveform, and (b), (c), (d), (e), and (f) are fault waveforms for* C1 *⇑,* C1 *⇓,* C2 *⇓,* R2 *⇓, and* R3 *⇓, respectively.

In order to close to the actual circuit characteristic, every fault class will be simulated 150 times in faulty parameter ranges using Monte Carlo analysis method in time domain and a total of 2250 corresponding impulse response waveforms are obtained. Some related waveforms are shown in Figure 5. In the figure, (a) is the fault-free waveform and others are different impulse response waveforms about different faulty circuits.

The simulation data in PSpice are recorded and imported into Matlab, and then their feature vectors are constructed with kurtosis and relative entropy to train an ELM classifier. The detail is described as follows.

**Figure 6:**
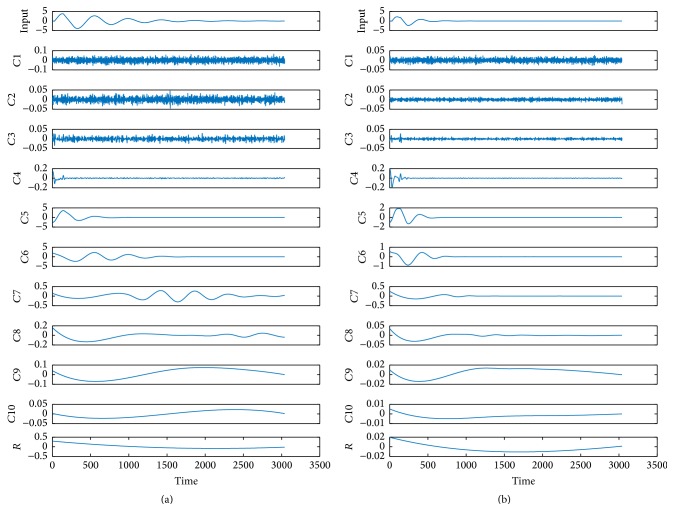
EEMD decomposition results of two response signals. In the decomposition, noise for standard deviation 0.2 is added, and the ensemble number is 800. (a) The nominal response signal decomposition results; (b) EEMD results of the response signal of* C1 *⇓.

First, to construct the feature vectors, we decompose these stored responses data into IMF components with EEMD method based on the discussion in Section 3.  Figure 6 displays the results of EEMD decomposition of no-fault circuit and faulty* C1 *⇓. In the figure, each of the two response signals is decomposed into 10 IMF curves and a residue from high frequency to low frequency. From the figure, we can clearly see that in same decomposition layer, faulty IMF differs obviously from fault-free IMF. Therefore, here, we only take* C4–C9* IMF components into account to improve fault distinguishability that can satisfy our work.

Next, kurtosis of each IMF for a certain fault circuit is calculated. Meanwhile each IMF waveform (300 *μ*s, 3000 samples) is averagely divided into 6 segments. The PDF of each IMF is calculated according to (12), (13), and (14).  Table 2 demonstrates PDFs of the nominal response IMFs for fault-free circuit. Therefore, relative entropy between each IMF of faulty components and corresponding nominal IMF of fault-free circuit is achieved by adopting formula (15). Feature vector *T* for each fault class will be built to feed directly into an ELM classifier. Take* C1 *⇓; for example, Table 3 shows a result of feature extraction which is a feature vector of the faulty* C1* through calculating its kurtosis and relative entropy with (11), (15), and (16). Its fault feature vector is *T* = [0.6162, 0.8729, 0.9813, 0.8713, 0.9203, 0.7989, 0.0099, 0.0109, 0.2260, 0.7400, 0.1266, 0.4903]. As the same way, a total of 2250 fault feature vectors of the circuit can be obtained.

Finally, for every fault class of the Sallen-Key circuit, 150 samples are split into two parts. The first 100 fault feature vectors are adopted to train an ELM classifier and the remaining 50 fault feature vectors are used to test the ELM. Because the testing accuracy is sensitive to the selection of activation functions, the RBF function is proper for the diagnostic and the number of neurons is set as 250.

In order to show the performance of the proposed diagnostic method, we compare our method with other existing feature extraction methods which are presented in [3, 11, 18] to train an ELM classifier. The results of classification are demonstrated in Table 4. For the single faults diagnosis of the Sallen-Key bandpass filter circuit, the average test accuracy of our method is 99.4%. In contrast, the wavelet and ELM method (88.8%), the lifting wavelet and ELM method (99.3%), and the method in [11] (97.9%) are lower than ours in test accuracy. Thus, we can see that the performance of the proposed method is superior to the combination method of wavelet and ELM and the method of [11]. Meanwhile, it has nearly the same accuracy as the lifting wavelet and ELM method. Moreover, these methods [3, 11, 18] only considered a CUT to be faulty when the value of potential faulty component is higher or lower than the nominal value by 50% and did not take the continuity of faulty parameters and the influence of tolerances into account. If we use the same method considering only 50% variation as faulty parameter values, the test accuracy of our method could be up to 100% in simulation.

For reducing time cost, we adopt an ELM algorithm as a classifier because it is one of the best classification algorithms and it also can provide higher performance in time cost.  Table 5 shows ELM-based method's performance and SVM-based method's performance in time cost with the same four types of original feature vectors, respectively. The four feature vectors are based on different feature extraction methods. From the table, it can be seen that the test accuracy of ELM-based method is approximate to SVM-based method's accuracy. For example, the test accuracy of SVM-based method is 98.6% for the kurtosis and entropy technique, which is similar to the ELM-based method (97.9%). However, the time consumption of the ELM-based method is much lower than the SVM-based method. For instance, using wavelet coefficients as features to train SVM classifiers, it takes 11.2 s; on the contrary, it takes 0.0289 s with an ELM classifier. For our proposed method, its time cost is better than wavelet coefficients technique and lifting wavelet technique. As a result, the proposed method in the paper can reduce time cost greatly.

### 4.2. A Leapfrog Filter

**Figure 7:**
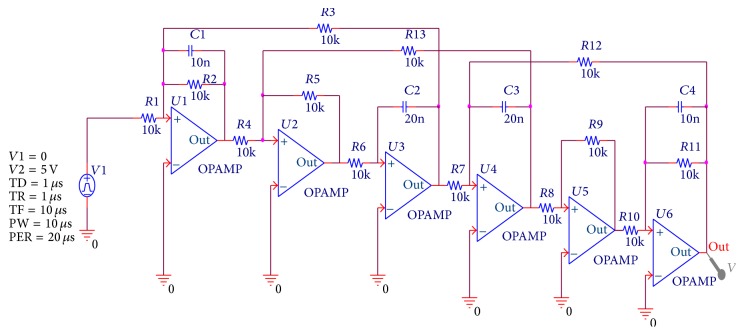
Schematic of a leapfrog filter.

The second example circuit is a leapfrog filter, which is used as a CUT in [9]. The nominal values of the benchmark circuit's components are shown in Figure 7. The input signal is also a single pulse with 5 V amplitude and 10 *μ*s duration. The “out” point of the circuit is the only test point. As we all know, several components of a CUT may cause faults simultaneously in practice. Therefore, in the experiment, 10 multifault cases are selected to verify our proposed method's diagnostic performance for multifaults in the CUT, which is the same fault option as in [9]. These fault classes are shown in Table 6. The experiment is also carried out through injecting these faults classes to the CUT, respectively, according to the diagnostic procedure already discussed in Section 3. Diagnostic results of the circuit for multifaults are shown in Table 7. In the table, the average test accuracy of our method is 98.9%, whereas for these methods adopting these feature extraction methods in [3, 11, 18] to diagnose these multifaults in the leapfrog filter circuit their diagnostic accuracies are 88.1%, 90.5%, and 86.8%, respectively. Therefore, we can see that the proposed method is better than the other diagnostic methods for multifaults in the leapfrog filter circuit.

Through the two experiments, the results of the proposed method can be summarized as follows:

1. The proposed method in the paper has better accuracy than other methods such as the first wavelet coefficients technique and the lifting wavelet method.
2. For multifaults diagnosis, the method adopting EEMD, kurtosis, and relative entropy to construct feature vectors has better classification accuracy than the traditional method used in [3, 11, 18].
3. ELM classifiers with the techniques of EEMD, kurtosis, and relative entropy sometimes get the same better classification results as SVM classifiers with the same original feature vectors. Meanwhile, ELM-based method has much lower classification time than SVM-based method.

To sum up, the proposed method in the paper is acceptable from two aspects: test accuracy and time cost. It has higher test accuracy and fast classification capacity.

## 5. Conclusions

In this paper, a combinational diagnostic method for analog circuit with EEMD, relative entropy, and ELM is proposed. The proposed method makes good use of the EEMD, kurtosis, and relative entropy technique to construct fault feature vectors, and then faults classification on CUTs are performed using the ELM classifier. The effectiveness of the proposed method has been validated with the classical two benchmark circuits for single and multifault diagnosis. The results of experiments show that the method can distinguish effectively different faults of circuit with the higher testing accuracy (99.4%) and the lower testing time.

## Figures and Tables

**Table 1:** Fault configuration for Sallen-Key filter circuit.

Fault ID	Faults	Nominal values	Faulty parameter ranges
F0	No-fault		
F1	*R1 *⇓	5.18k	[0.5, 4.92k]
F2	*R1 *⇑	5.18k	[5.44k, 51meg]
F3	*R2 *⇓	1k	[0.1, 0.95k]
F4	*R2 *⇑	1k	[1.05k, 10meg]
F5	*R3 *⇓	2k	[0.2, 1.9k]
F6	*R3 *⇑	2k	[2.1k, 20meg]
F7	*R4 *⇓	4k	[0.4, 3.8k]
F8	*R4 *⇑	4k	[4.2k, 40meg]
F9	*R5 *⇓	4k	[0.4, 3.8k]
F10	*R5 *⇑	4k	[4.2k, 40meg]
F11	*C1 *⇓	5n	[0.5p, 4.75n]
F12	*C1 *⇑	5n	[5.25n, 50u]
F13	*C2 *⇓	5n	[0.5p, 4.75n]
F14	*C2 *⇑	5n	[5.25n, 50u]

**Table 2:** PDF of the nominal IMFs of fault-free circuit.

Fault name	IMFs	PDF					
		*p*_*n*1_	*p*_*n*2_	*p*_*n*3_	*p*_*n*4_	*p*_*n*5_	*p*_*n*6_
Fault-free	*C4*	0.9710	0.0061	0.0047	0.0058	0.0063	0.0060
	*C5*	0.9683	0.0316	0.0000	0.0000	0.0000	0.0000
	*C6*	0.4853	0.4481	0.0657	0.0009	0.0000	0.0000
	*C7*	0.1316	0.0892	0.3222	0.3515	0.0982	0.0073
	*C8*	0.5588	0.3277	0.0419	0.0011	0.0228	0.0476
	*C9*	0.1302	0.2072	0.0276	0.2257	0.3032	0.1062

**Table 3:** Feature vector of *C1 *⇓.

Fault name	IMFs	Relative entropy *D*_*i*_	Normalized kurtosis	Feature vector *T*
*C1 *⇓	*C4*	0.0099	0.6162	*T*_norm_ = [0.6162 0.8729 0.9813 0.6713 0.9203 0.7989 0.0099 0.0109 0.2660 0.7400 0.1266 0.4903]
	*C5*	0.0109	0.8729	
	*C6*	0.2660	0.9813	
	*C7*	0.7400	0.8713	
	*C8*	0.1266	0.9203	
	*C9*	0.4903	0.7989	

**Table 4:** Results of fault classification of the Sallen-Key filter for single faults.

Fault number	Fault name	Test accuracy			
		Proposed	Wavelet [3] + ELM	Lifting wavelet [18] + ELM	[11] + ELM
F0	No-fault	1.0000	0.8600	1.0000	1.0000
F1	*R1 *⇓	0.9700	0.9800	1.0000	0.9800
F2	*R1 *⇑	1.0000	0.8700	1.0000	1.0000
F3	*R2 *⇓	1.0000	1.000	1.0000	0.9300
F4	*R2 *⇑	1.0000	0.9800	1.0000	1.0000
F5	*R3 *⇓	1.0000	0.8700	1.0000	1.0000
F6	*R3 *⇑	0.9700	0.7800	0.9500	0.8200
F7	*R4 *⇓	0.9900	0.9400	1.0000	1.0000
F8	*R4 *⇑	1.0000	0.8400	1.0000	1.0000
F9	*R5 *⇓	1.0000	0.9600	1.0000	1.0000
F10	*R5 *⇑	1.0000	0.8200	1.0000	1.0000
F11	*C1 *⇓	1.0000	0.8000	1.0000	1.0000
F12	*C1 *⇑	0.9700	0.6400	0.9400	0.9600
F13	*C2 *⇓	1.0000	1.0000	1.0000	1.0000
F14	*C2 *⇑	1.0000	0.9800	1.0000	1.0000
Average accuracy		0.9940	0.8880	0.9930	0.9790

**Table 5:** Comparison of time cost and test accuracy between ELM-based method and SVM-based method for the Sallen-Key bandpass filter.

Feature extraction method	Time (s)/accuracy (%)	
	SVM classifier	ELM classifier
Wavelet coefficients [3]	11.2/93.1	0.0289/88.8
Kurtosis and entropy [11]	7.1/98.6	0.0213/97.9
Lifting wavelet [18]	14.6/99.2	0.0350/99.3
Proposed method	9.3/99.8	0.0275/99.4

**Table 6:** Fault classes of the leapfrog filter for the multifaults.

Fault ID	Nominal value	Fault value
F1	*R2*: 10k, *R4*: 10k	*R2*: 20k, *R4*: 20k
F2	*R2*: 10k, *R4*: 10k	*R2*: 5k, *R4*: 5k
F3	*R3*: 10k, *R4*: 10k	*R3*: 20k, *R4*: 5k
F4	*R3*: 10k, *R4*: 10k	*R3*: 5k, *R4*: 5k
F5	*R4*: 10k, *R8*: 10k	*R4*: 15k, *R8*: 5k
F6	*R4*: 10k, *R8*: 10k	*R4*: 5k, *R8*: 20k
F7	*C2*: 20nf, *C3*: 20nf	*C2*: 30nf, *C3*: 30nf
F8	*C2*: 20nf, *C3*: 20nf	*C2*: 10nf, *C3*: 10nf
F9	*C2*: 20nf, *C4*: 10nf	*C2*: 10nf, *C4*: 20nf
F10	*C2*: 20nf, *C4*: 10nf	*C2*: 10nf, *C4*: 5nf

**Table 7:** Diagnostic results of the leapfrog filter for the multiparametric faults.

Diagnostic method	Fault ID										Average accuracy (%)
	F1	F2	F3	F4	F5	F6	F7	F8	F9	F10	
Reference [3]	100	100	51	46	86	100	100	100	98	100	88.1
Reference [11]	100	70	100	98	70	74	100	100	95	98	90.5
Reference [18]	100	100	52	34	82	100	100	100	100	100	86.8
Our method	100	98	100	100	94	96	100	100	100	100	98.8
